# MicroRNA-Mediated Restriction of HIV-1 in Resting CD4^+^ T Cells and Monocytes

**DOI:** 10.3390/v4091390

**Published:** 2012-08-29

**Authors:** Karen Chiang, Andrew P. Rice

**Affiliations:** 1 Interdepartmental Program in Translational Biology and Molecular Medicine, Houston, TX 77030, USA; Email: k5chiang@ucsd.edu (K.C.); arice@bcm.edu (A.R.); Tel.: +1-713-798-5774 (A.R.); Fax: +1-713-798-3170 (A.R.); 2 Molecular Virology and Microbiology, Baylor College of Medicine, Houston, TX 77030, USA

**Keywords:** miRNAs, HIV, CD4^+^ T cells, monocytes/macrophages

## Abstract

In contrast to activated CD4^+^ T cells and differentiated macrophages, resting CD4^+^ T cells and monocytes are non-permissive for HIV-1 replication. The mediators which regulate the resting or quiescent phenotype are often actively involved in the restriction of viral replication and the establishment and maintenance of viral latency. Recently, certain microRNAs which are highly expressed in resting cells have been implicated in this capacity, inhibiting the expression of cellular proteins that are also viral co-factors; following activation these microRNAs exhibit decreased expression, while their targets are correspondingly up-regulated, contributing to a favorable milieu for virus replication. Other microRNAs exhibiting a similar expression pattern in resting and activated cells have been shown to directly target the HIV-1 genome. In this review we will discuss the resting state and the causes behind viral restriction in resting cells, with emphasis on the role of microRNAs.

## 1. CD4^+^ T Cells

### 1.1. T Cell Activation

**Figure 1:**
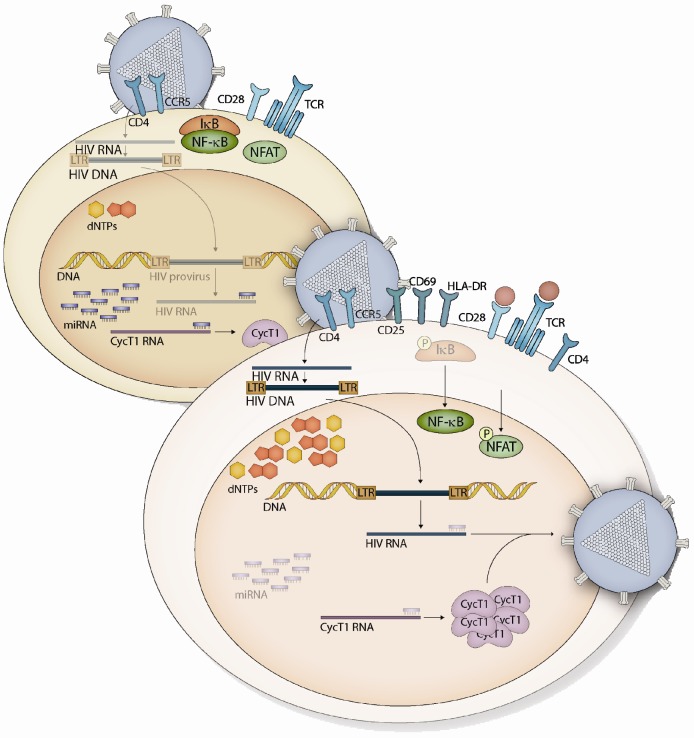
Mechanisms of HIV-1 restriction in resting CD4^+^ T cells and HIV-1 permissivity in activated CD4^+^ T cells. HIV-1 infection is blocked at several early steps in resting CD4^+^ T cells, including reverse transcription, integration, and proviral transcription. Low nucleotide levels and cytoplasmic sequestration of key factors which mediate proviral transcription both contribute to restriction. Furthermore, miRNAs which target HIV-1 RNA are expressed at high levels in resting cells, along with miRNAs which repress translation of the Tat co-factor Cyclin T1. Following activation, in this case depicted as engagement of the TCR and CD28 receptor, IκB is phosphorylated and degraded, freeing NF-κB to translocate to the nucleus. NFAT phosphorylation also results in its nuclear re-localization. Nucleotide levels undergo an increase, and reverse transcription of the HIV-1 genome proceeds efficiently, as does integration into host chromosomal DNA, and transcription of the provirus. This latter process is aided by increased levels of Cyclin T1, as Cyclin T1-targeting miRNAs (and HIV-targeting miRNAs) are downregulated after activation.

To fully understand the resting state of CD4^+^ T cells, it is perhaps useful to first discuss its perturbation by activation. Activation of naive CD4^+^ T cells is the first step in the differentiation of T helper (Th) cells into effector or regulatory subsets, and resting memory CD4^+^ T cells also undergo the activation process after stimulation by their cognate antigens. Full T cell activation requires two signals: T cell receptor (TCR) binding of a peptide-MHC complex presented on the surface of an antigen-presenting cell (APC), along with a costimulatory signal delivered via the CD28 receptor [1]. In the absence of the costimulatory signal, T cell anergy ensues, a state of antigen hyporesponsiveness involved in the development of tolerance, or discrimination of self antigens [2]. TCR ligation results in phosphorylation of the cytoplasmic tail of the adjacent CD3 complex, which then recruits the LAT and SLP-76 adapter proteins [3]. The adapters organize the assembly of a proximal signaling complex comprising numerous effector kinases, which enable the triggering of multiple signaling cascades that eventually result in the nuclear accumulation of the transcription factors NFAT, NF-κB, and AP-1. For instance, activation of PLCγ1 results in the production of second messengers IP_3_ and DAG; DAG production activates a MAPK signaling pathway via Ras which culminates in activation of the AP-1 complex. DAG also contributes to the activation of PKCq, resulting in the phosphorylation and degradation of the inhibitor of NF-κB (IκB) by the IκB kinase (IKK) complex, enabling nuclear translocation of NF-κB (Figure 1). IP_3_ stimulates the mobilization of intracellular Ca^2+^, activating calcium-dependent signaling proteins like calcineurin, which dephosphorylates members of the NFAT family, resulting in their nuclear translocation [3]. 

Synergistic interactions between NFAT, NF-κB, and AP-1 results in the upregulation of hundreds of genes, enabling clonal cell proliferation and cytokine production. This process occurs much more quickly in the case of memory CD4^+^ T cell activation, as these cells are primed for rapid antigen response [4]. Activation markedly upregulates the expression of IL-2, which is essential for T cell proliferation and survival [5]. During CD8^+^ T cell activation in mice, it has also been shown that there is a marked shift towards alternative upstream 3'UTR usage, which has been linked to escape from miRNA-mediated silencing [6]. This suggests that miRNAs in general exert a powerful repressive effect on protein translation in the resting state.

### 1.2. Resting CD4^+^ T Cells

While activated CD4^+^ T cells are the primary hosts to ongoing HIV-1 replication *in vivo*, the majority of CD4^+^ T cells (~95%) found in the human body are in the resting state, and resting memory CD4^+^ T cells comprise the main reservoir of latently infected cells [7]. The resting or quiescent phenotype in CD4^+^ T cells is typically defined experimentally by the maintenance of cells in the G_0_ phase of the cell cycle, and by the absence of activation marker expression, most commonly CD25, CD69, and HLA-DR. Activated cells are also much larger in size than resting cells.

It is increasingly understood that quiescence is not simply a default state, but one which is actively maintained on several fronts [8]. Among others, the transcription factors LKLF, Tob, Foxo3a, and Foxj1 have been implicated in controlling gene expression in the resting state. LKLF appears to be a master regulator of quiescence, as its expression in Jurkat cells decreases cell proliferation and metabolic rate [9,10], and LKLF-deficient mice lack circulating CD4^+^ T cells, while the few T cells to be found in the lymph nodes display a spontaneously activated phenotype [11]. Tob represses the expression of various genes involved in cell cycle progression as well as IL-2 [12]. Foxo3a and Foxj1, two members of the forkhead family of transcription factors, are negative regulators of NF-κB which promote the expression of IκB [12]. All four transcription factors discussed above are down-regulated upon T cell activation [11,12].

Keeping in mind that proper immune system function depends on a timely response to activating signals, particularly in the case of rapidly acting memory T cells [4], it is perhaps more useful to think of the resting state not simply as a quiescent shut-down, but rather as a state of expectant readiness. While global RNA transcription is decreased in resting cells, it appears that many genes induced upon activation display the chromatin modifications associated with active genes, despite their silenced state in resting cells [13,14,15]. This suggests that these genes, which include some coding for miRNAs, are primed for activation, and indeed RNAP II has been found to be poised at the promoters of these genes, paused in a promoter-proximal position. This stalling of RNAP II, now understood to be a commonly utilized mechanism of gene regulation [16], is thought to occur because the RNAP II complex initiates transcription yet is non-processive for transcriptional elongation, which is dependent on the recruitment of the cellular complex positive transcription elongation factor b (P-TEFb). P-TEFb is a heterodimer of Cdk9 and Cyclin T1 or T2, and protein levels of Cdk9 and Cyclin T1, which is found in the majority of P-TEFb complexes, are low in resting CD4^+^ T cells [17]. This state of affairs is quickly reversed upon cellular activation, thereby allowing for transcriptional elongation of poised genes. Cyclin T1-containing P-TEFb is also a key mediator of the viral protein Tat, as Tat-mediated recruitment of P-TEFb is essential for proviral transcription [18].

Activation also increases the nuclear localization of NF-κB, NFAT, and AP-1, which mediate transcription of the provirus. Nucleotide availability is low in resting CD4^+^ T cells [19,20], and nucleotide requirements are primarily met by salvage pathways, not via *de novo* synthesis [21,22]. Protein synthesis is also lower in resting cells [8], and overall a catabolic metabolism prevails, where ATP is generated from biosynthetic precursors like glucose via the TCA cycle and oxidative phosphorylation, supplying the minimum of energy needed to maintain housekeeping functions [23,24].

### 1.3. HIV-1 Restriction in Resting CD4^+^ T Cells

HIV-1 infection of resting CD4^+^ T cells is nonproductive due to multiple blocks in the viral life cycle. Intriguingly, activation of resting cells only two hours post-infection fails to rescue viral replication to the levels exhibited by cells stimulated prior to infection, suggesting that restriction of early events in resting cells limits viral replication [25], although it is unclear what exactly they are. It is known that reverse transcription occurs much more slowly in resting cells [26,27], and while the accumulation of incomplete and full-length reverse transcripts can be observed [28,29,30], the slow kinetics of reverse transcription probably render these and other viral components highly susceptible to decay mechanisms, reducing the likelihood of integration [30]. The inefficiency of reverse transcription may be due to the low availability of free nucleotides in resting cells [29,31], or to as yet undiscovered mechanisms, as nucleotide supplementation fails to fully rescue viral cDNA production or replication kinetics to the levels seen in activated cells [30,31,32]. Integration does occur in resting CD4^+^ T cells [33,34], although inefficiently, with abnormal integration events and increased production of abortive forms like 2-LTR circles [35]. Interestingly, the frequency of integration into active sites of transcription for resting CD4^+^ T cells was comparable to that of activated CD4^+^ T cells, despite the expected decrease in chromatin access in the resting state [35]. Restriction factors in resting CD4^+^ T cells may also inhibit productive HIV-1 replication [36]. One report has shown that siRNA knockdown of Murr1, an inhibitor of NF-κB activity, increased HIV-1 replication in primary resting CD4^+^ T cells [37]. 

As the CD4 T cell count in peripheral blood is integral to the clinical monitoring of HIV-1 infection, it often goes unappreciated that the vast majority of CD4^+^ T cells are found in lymphoid tissues. In contrast to resting CD4^+^ T cells in the periphery, these cells can undergo productive HIV-1 infection. Resting CD4^+^ T cells in human tonsil explants undergo productive HIV-1 infection, in a manner dependent on the presence of the tissue microenvironment [38,39]. Resting CD4^+^ T cells in lymph nodes are a major source of virus replication during acute infection with SHIV [40], and mucosal memory CD4^+^ T cells in the macaque colon displaying a typical resting phenotype, CD25^-^CD69^-^Ki67^-^, have been shown to be productively infected by SIV [41]. The authors of the latter study speculated that these memory cells were not truly quiescent, perhaps having just recently returned to the resting state and thus still retaining sufficient nucleotide levels and transcription factor activity to support productive infection. Therefore these cells would not be considered completely quiescent, and it was observed that activated cells in the same tissue produced more virus. 

The infection of resting and activated CD4^+^ T cells in lymphoid tissues cannot completely account for the massive depletion of mucosal CD4^+^ T cells, particularly in the gut-associated lymphoid tissue (GALT), which occurs shortly after initial infection, as only a small minority of the killed cells are productively infected. While virus infection is directly cytopathic, it appears that infection also induces substantial bystander apoptosis of uninfected adjacent cells [42,43]. In *ex vivo* human tonsil cultures infected with HIV-1, it was shown that over 95% of the dying CD4^+^ T cells were bystanders, with the vast majority being resting CD4^+^ cells which had undergone abortive infection, inducing cell death [44]. As only ~5% of the CD4^+^ T cells were productively infected, this suggests that while a small minority of resting cells in lymphoid tissues may be productively infected, most are in fact non-permissive for viral replication. 

The resting CD4^+^ T cell, or more specifically, the resting memory CD4^+^ T cell, is also host to latent HIV-1 provirus. The generally accepted model for the establishment of a latently infected reservoir of CD4^+^ T cells suggests that activated cells are infected during their transition into memory CD4^+^ T cells [7]. Most CD4^+^ T cells activated in response to antigen will die within a couple days, but a select few survive and return to a resting state as memory CD4^+^ T cells, which are also nonpermissive for viral replication, thus prompting any newly integrated virus to enter latency. More specifically, two subtypes of memory T cells have recently been implicated in HIV latency: the central memory and transitional memory T cells [45]. It is thought that both the long half-lives of central memory cells and homeostatic proliferation that transitional memory T cells normally undergo for self-maintenance contribute to the persistence of the memory CD4^+^ T cell latent reservoir [46]. The maintenance of latency is intimately associated with the resting state, as exit from latency occurs when a memory cell encounters its specific antigen or following cytokine stimulation, leading to cell and virus activation. 

## 2. Monocytes and Macrophages

According to the classical model, monocytes function in innate immunity by circulating in the periphery, and upon detection of inflammatory signals, or during routine homeostatic maintenance, extravasate into tissues, where they can differentiate into dendritic cells and macrophages [47]. Macrophages are resident in tissues, where they phagocytose pathogens, present antigens, and produce inflammatory cytokines to recruit additional immune cell help [48]. Both monocytes and macrophages can be divided into further subsets with varying functions, and not all macrophages derive from monocytes [49]. However, as HIV experiments have mostly been conducted in monocyte-derived macrophages (MDMs), owing to the difficulties in working with tissue macrophages, for the purposes of this review we will consider monocytes and macrophages as single classes. 

### Monocyte and Macrophage Restriction of HIV-1 Replication

Unlike activated CD4^+^ T cells, both monocytes and monocyte-derived macrophages are typically considered to be steady state non-proliferating cell types. However, in parallel with resting CD4^+^ T cells, monocytes are generally considered non-permissive for viral replication, while permissivity in macrophages is greatly increased [50]. The various blocks to replication which exist in monocytes and to some extent in macrophages has been nicely reviewed elsewhere [51], but we will provide a summary here. Higher levels of CCR5 expression have been shown to correlate with increased viral replication [52,53], but inefficient viral entry cannot completely account for low permissivity, as it has also been shown that VSV-G pseudotyped virus replicates quite poorly in monocytes [30,54]. However, even VSV-G pseudotyped particles appear to undergo a block during entry, as intracellular levels of p24 measured 2 hours post-infection quickly plateau at a low level, becoming unresponsive to increasing amounts of virus [55]. Pseudotyped virus expressing Vpr-beta-lactamase (BLAM) also showed a defect in entry, as measured by decreased cleavage of intracellular CCF2 dye [55,56]. In addition to a possible entry block, HIV-1 infection in monocytes also appears to be hindered at the level of reverse transcription, which proceeds very slowly compared to rates in macrophages [54,55,57]. This may be due to low nucleotide availability [58], as has also been suggested for T cells. On the other hand, it has been demonstrated that an HIV-based lentiviral vector undergoes efficient reverse transcription in monocytes, but fails to produce 2-LTR circles, indicating a block prior to integration [59]. Blocks also exist at the level of transcription, as co-nucleofection of an LTR-luciferase reporter plasmid and pTat did not result in any detectable luciferase expression, indicating that Tat’s transactivation ability is restricted in monocytes [60]. While this is likely due in part to low, almost undetectable levels of Tat co-factor Cyclin T1 in monocytes [61], Cyclin T1 protein being induced upon HIV-1 infection of macrophages [62], a modest increase in protein levels following nucleofection of pCyclin T1 cannot restore Tat transactivation [60], suggesting additional blocks to transcription (and/or a very high limiting level of Cyclin T1 in monocytes). 

While monocytes are generally non-permissive for HIV-1 infection, replication competent virus can be recovered following activation of monocytes from HIV-infected patients [63,64], and a small population of monocytes appear to be undergoing active infection, even in the presence of suppressive HAART [65]. This may represent another reservoir of latent virus, although the frequency of monocytes harboring HIV-1 DNA appears to be very low, at <0.1% of total monocytes [66,67]. As HIV-1 DNA has been found in CD34^+^ hematopoietic progenitor cells (HPCs) isolated from aviremic patients and a low percentage of HPCs are susceptible to HIV-1 infection *ex vivo* [68], it is also possible that the differentiation of these cells eventually gives rise to infected monocytes. However, the ability of HIV-1 to establish latency in these precursor cells is still controversial, as two other groups did not detect any proviral DNA in all patients examined [69,70], and only 28 individuals in total have been studied to date.

Although macrophages are much more permissive to HIV-1 infection and replication, susceptibility to infection can vary by up to 3,000-fold in MDMs prepared from different donors [71,72,73], while in identical twins replication efficiency and kinetics are similar [71], strongly suggesting the influence of host genetic factors. Furthermore, it appears that not all MDMs are able to replicate virus, as cytokine-induced polarization can greatly affect susceptibility to infection [74,75]. Restriction in MDMs can be relieved by expression of the Vpx gene of HIV-2/SIV [76], and it has recently been discovered that Vpx counteracts the restriction factor SAMHD1, which appears to block viral replication at the reverse transcription step [77,78]. Further work has shown that SAMHD1 is an enzyme which breaks down dNTPs [79,80], and supplying exogenous dNTPs to MDMs can substitute for Vpx in promoting virus replication [81]. 

## 3. miRNAs and HIV-1 Replication in Resting CD4^+^ T Cells and Monocytes

### 3.1. miRNA Expression Contributes to HIV-1 Restriction

In a seminal paper, the Benkirane lab demonstrated that knockdown of the miRNA processing enzymes Dicer and Drosha increases HIV-1 replication [82], suggesting that the miRNA pathway in general represses HIV-1 replication and also contributes to the maintenance of latency, as Drosha silencing also re-activated virus from latently infected PBMCs isolated from patients on suppressive HAART [83]. Profiling studies have generally shown that miRNAs downregulated upon T cell activation outnumber the upregulated miRNAs, indicating increased target de-repression that is consistent with the general growth-conducive phenotype established by activation [84,85]. Multiple studies have thus employed comparisons of miRNA expression in resting and activated CD4^+^ T cells to identify miRNAs which can affect viral replication, reasoning that miRNAs down-regulated upon activation may be helping to mediate HIV-1 restriction in resting cells [86,87,88]. While direct comparison between miRNA expression profiling experiments is complicated by differing experimental methodologies, a few miRNAs have been found to be consistently downregulated in numerous studies and capable of repressing viral replication (Table 1). 

One such miRNA is miR-150, which exhibits a highly lymphopoietic-specific expression pattern, and is found at high levels in mature and resting B and T cells [89,90,91]. Initial studies on miR-150 showed that its overexpression or ablation led to defects in murine B cell development, and it was shown to target the transcription factor c-Myb [90,91]. More recent work has shown that T cell development and differentiation is also highly dependent on miR-150; while precursor cells have low levels of miR-150, resting mature cells have high levels, which decrease following activation or differentiation [89,91]. In T cells, while c-Myb also appears to be important for lymphopoiesis, miR-150 was shown to target NOTCH3 and overexpression of miR-150 interferes with cell proliferation in T cell lines [92]. In 2007, miR-150, along with miR-28, 125b, 223, and 382, was shown to target the 3'UTR region common to almost all HIV-1 mRNA transcripts [86]. While transfection of individual antagomiRs to these miRNAs modestly increased virus production in resting CD4^+^ T cells transfected with pNL4.3, transfection of a cocktail of all five antagomiRs led to a significant increase in viral output. Furthermore, transfection of the antagomiR cocktail re-activated latent virus from resting CD4^+^ T cells isolated from aviremic patients on suppressive HAART. Interestingly, it was also shown that all five of these HIV-targeting miRNAs were down-regulated following PHA activation of primary resting CD4^+^ T cells [86]. 

**Table 1:** MiRNAs down-regulated after CD4^+^ T cell activation or monocyte-macrophage differentiation. All miRNAs have also been shown to inhibit HIV-1 replication when overexpressed, and some have also been shown to undergo downregulation following *in vitro* HIV-1 infection. For other relevant targets, gene name followed by question mark indicates a possible indirect target.

*CD4^+^ T cells*	HIV-1 RNA target site	References showing downregulation after infection	Other relevant targets	Notes
**miR-17-92 cluster**	N	[82,93]	*P/CAF*	
**miR-27 (a, b)**	N	27a: [93,94]	miR-27b: *Cyclin T1*	Downregulated by *H. saimiri* and BLV
**miR-29 (a, b, c)**	Y	[93,94,95]	miR-29b: *Cyclin T1* (?)	Targets HIV RNA to RISC and P-bodies [96]
**miR-150**	Y	[93]	*c-Myb, Notch3, Cyclin T1* (?)	Mediates T cell differentiation
**miR-223**	Y	[93], upregulated in: [95]	*Cyclin T1* (?)	Also decreased during monocyte-macrophage differentiation
***Monocytes***				
**miR-198**	N	Upregulated after CEMx174 infection: [94]	*Cyclin T1*	Appears to be differentially regulated in CD4^+^ T cells *vs.* macrophages

Our own work has confirmed the downregulation of these miRNAs in response to PHA, and has further shown that some of these same miRNAs plus other miRNAs down-regulated after cell activation target the Tat co-factor Cyclin T1. In resting CD4^+^ T cells and monocytes, Cyclin T1 protein expression is very low, and is dramatically induced upon activation or differentation in a manner independent of a relative increase in Cyclin T1 mRNA [61,97,98,99]. This strongly suggested that Cyclin T1 is under post-transcriptional repression in resting cells, and indeed we have found several miRNAs involved in this process. Comparing the miRNA expression profile of monocytes to macrophages, we found miR-198 to be significantly down-regulated upon differentiation, and showed that miR-198 overexpression decreased Cyclin T1 protein levels [87]. We also identified a miR-198 binding site within the Cyclin T1 3'UTR using 3'UTR:luciferase assays and mutational analysis for confirmation. Transduction with miR-198-encoding lentivirus increased virus production in MM6 cells, a promonocytic cell line. In CD4^+^ T cells, miR-198 is expressed at very low levels and does not undergo a significant downregulation after activation; instead, we have identified several miRNAs which appear to be repressing Cyclin T1 protein in the resting state: miR-27b, miR-29b, miR-150, and miR-223 [88]. While we were able to experimentally validate a miR-27b binding site within the Cyclin T1 3'UTR, we were unable to confirm predicted binding sites for the other miRNAs. However, inhibition of both miR-27b and miR-29b reduced the association of Cyclin T1 mRNA with the RISC complex, as assayed by Ago2 immunoprecipitation, suggesting that both of these miRNAs are mediating silencing of the Cyclin T1 transcript. As mentioned above, miR-150 and miR-223 are known inhibitors of HIV-1 replication [86], as is miR-29b [96,100], which has also been shown to target the viral RNA. We showed that overexpression of miR-27b also acts to decrease viral replication, and in a Cyclin T1-dependent manner [88]. Another group has found yet another post-transcriptional mechanism of keeping Cyclin T1 protein levels low in monocytic cells, as a protein called NF90 suppresses Cyclin T1 translation in monocytes, but its expression is decreased following PMA activation, allowing for increased Cyclin T1 protein levels [101]. This further underscores the importance of Cyclin T1 protein regulation in monocytes.

These results indicate that miRNAs which negatively affect HIV-1 exert antiviral effects both via direct targeting of the HIV-1 RNA and targeting of cellular co-factors of virus replication (further reviewed in [102]), and are often down-regulated following CD4^+^ T cell activation or macrophage differentiation. This is unsurprising given the heavy dependence of efficient HIV-1 replication on cell activation and given that coordinately regulated miRNAs have been shown to act cooperatively in cellular pathways like activation, either via redundancy, where multiple miRNAs target the same gene, or via multiplicity, where a single miRNA targets multiple genes in the pathway [103]. In addition to miR-150, other miRNAs identified in these studies have also been associated with T cell differentiation; for example miR-125b overexpression inhibited differentiation of naive CD4^+^ T cells into effector cells, suggesting that miR-125b contributes to the maintenance of the resting state [85]. In macrophages, miR-125b appears to have another function, as overexpression increases responsiveness to IFN-γ and the ability to stimulate T cells following antigen exposure [104]. Expression of miR-28, 125b, 150, and 382 is also increased following IFN-α or -β treatment of primary monocytes [105]. Furthermore, it has been demonstrated that treatment of cells with antagomiRs against miR-28, 150, 223, and 382 increased the susceptibility of monocytes to HIV-1 infection, while transfection of the miRNAs into macrophages decreased viral replication [106]. 

### 3.2. Viral Infection Potentiates Changes in miRNAs Highly Expressed in Resting Cells

While the anti-HIV-1 miRNAs thus far discussed are downregulated upon cell activation or differentiation, substantially neutralizing their ability to inhibit viral replication, virus infection itself can also lead to changes in miRNA expression. The well-defined miR-17-92 cluster is downregulated following HIV-1 infection of Jurkat cells, and was subsequently shown to target P/CAF, a histone transacetylase which serves as a Tat co-factor [82]. Overexpression of two members of the cluster, miR-17-5p or miR-20a, decreased viral replication. Other studies have shown that T cell activation also decreases levels of most miRNAs in the miR-17-92 cluster [93].

Studies have also characterized miRNA expression patterns in cells from HIV-infected patients and in cells infected *ex vivo* with virus. In the first of these profiling studies, 62 miRNAs were found to be dysregulated by at least two-fold when compared to PBMCs from uninfected controls; the vast majority of the miRNAs were down-regulated in infected patients, as only three miRNAs were found to be differentially upregulated. Further stratification of patients into four classes: high CD4^+^ T cell count (>350)/low viral load (VL<400), high CD4/high VL (>10,000), low CD4 (<200)/low VL, and low CD4/high VL demonstrated that each class had its own distinguishing miRNA signature [93]. Out of the 59 miRNAs downregulated in all four patient classes, it was found that 37 (~63%) of these were also downregulated following anti-CD3 stimulation of PBMCs. MiR-150 and the miR-29 family members were among the down-regulated miRNAs, and were also recently confirmed by an independent group to be downregulated in the presence of viremia [107]. However, it was observed that 50% or more of the miRNAs exhibited differential regulation in PBMCs infected *ex vivo* with NL4-3 when compared to PBMCs isolated from infected patients [93], and only 32 of the 59 miRNAs (~54%) downregulated in patients exhibited a similar decrease in the T cell line CEMx174 after NL4-3 infection [94]. A simple comparison of two different studies in which PBMCs were infected with the HIV-1 strains NL4-3 or IIIB indicated that miR-29a and miR-29b were the only two miRNAs to be significantly down-regulated in both studies [93,95]. Although this kind of analysis is considerably complicated by differences in definitions of significance and experimental set-ups, particularly elapsed time between infection and harvest and cytokine treatment of PBMCs, the differences are nevertheless striking. More direct comparisons between *in vitro* infections of PBMCs and T cell lines have also shown considerable disparities between these cell types [95], emphasizing a need for conducting future studies in primary cells whenever possible, particularly purified CD4^+^ T cells and macrophages.

There is also evidence to suggest that other viruses have evolved similar relationships with these miRNAs. The miR-27 family (miR-27a and miR-27b, differing in only one nucleotide at the 3' end) has also recently been implicated in the replication process of two viruses found in New World primates and mice. *Herpesvirus saimiri* expresses a non-coding RNA, HSUR1 (*H. saimiri* U-rich RNA 1), which down-regulates miR-27 expression, resulting in low levels of miR-27 in herpesvirus-transformed marmoset T cells [108]. MiR-27 is also down-regulated following murine cytomegalovirus (MCMV) infection of cell lines and *in vivo* by a viral transcript which acts as a miR-27b target and mediates its degradation by the addition of post-transcriptional modifications [109,110,111]; over-expression of miR-27 leads to greatly decreased viral replication, while disruption of its target site in the viral transcript attenuates viral infection in mice. It is currently unknown which targets of miR-27 drive these two viruses to decrease levels of miR-27 as a means of increasing protein expression of critical viral co-factors. While it is very likely that a variety of miR-27 targets contribute to viral replication, we suggest that increases in Cyclin T1, as a transcriptional elongation factor, would be beneficial for supporting general viral replication. The bovine leukemia retrovirus (BLV) encodes a miR-29 analog (the miR-29 family encompasses three members: miR-29a, b, and c, which have the same seed region), which has been suggested to contribute to B cell tumorigenesis [112]. This adoption of a host strategy by a virus indicates the critical importance of miRNAs in regulating lymphocyte status and proliferation.

## 4. Perspectives

Much has been elucidated in the last few years with regards to the regulation of miRNAs in resting CD4^+^ T cells and monocytes, and their effects on HIV replication. However, there is also an increasing need for non-biased and genome-scale methods to keep pace with the rapid progress being made in the field. Novel mechanisms of miRNA-mediated regulation and of the regulation of miRNAs themselves have come to light; for instance mounting evidence indicates widespread reciprocal regulation between miRNAs and target mRNAs [113]. Base-pairing between target sequence and miRNA can result in 3' end trimming of the miRNA and non-specific addition of nucleotides, which can lead to miRNA decay [114,115,116]. RNA editing or differential miRNA processing likely also gives rise to so-called isomiRs, or miRNA variants initially dismissed as artifacts of deep-sequencing studies, but which appear to extend the targeting range of a single miRNA to other components of the same pathway [117]. Single miRNA-single gene studies may be pushed to the wayside as new methods to define all of the transcripts targeted by an individual miRNA or miRNA cluster have emerged, such as HITS-CLIP (high-throughput sequencing of RNAs isolated by crosslinking immunoprecipitation) and PAR-CLIP (which employs a photo-activatable ribonucleoside analog to identify cross-linked RNA), which immunoprecipitate Ago2 or other components of miRNA-containing ribonucleoprotein complexes (miRNPs) and deep sequence the associated miRNAs and mRNAs [118,119]. As RNase-treatment is used to define miRNP-protected sites, these methods return a relatively short length of bound target sequence, greatly assisting in target site identification. Yet mRNA targeting does not necessarily lead to decreases in protein expression, which can be examined on a genome-wide scale with mass spectrometry-based methods [120,121]. Ribosome profiling has also been used to determine if RISC-targeted mRNAs actually undergo translational inhibition [122,123]. Although these methods are very resource- and training-intensive, when combined with network analysis, their potential in elucidating HIV’s effects on miRNA expression and vice versa is extremely promising.

The cellular context of infection is one of the primary determinants of a successful outcome, and the cell type-specific expression or activity of miRNAs must be considered in the relevant environment. Individual miRNAs may be downregulated upon macrophage differentiation but remain unchanged or increased following CD4^+^ T cell activation, as is the case for miR-198 [87]. While miR-198 represses Cyclin T1 protein levels in monocytes, this function appears to be supplied by miR-27b, 29b, 150, and 223 in resting CD4^+^ T cells, suggesting that Cyclin T1 suppression is important in both cell types, although each has employed different miRNA expression patterns to achieve the same goal. This is also very likely to be the case across CD4^+^ T cell subsets; evidence from murine CD8^+^ T cells indicates that miRNA expression levels range from low in effector cells, higher in memory cells, and highest in naive cells [84]. Identifying distinct miRNA expression patterns in central memory CD4^+^ T cells may be of particular interest, as these cells appear to harbor the majority of latent infections in human patients on suppressive HAART [45], and miRNA-regulated targets in resting memory cells may contribute to the maintenance of latency. 

Profiling of miRNAs in infected individuals has been useful in identifying consistently dysregulated miRNAs, although there is often much disparity with those altered following *in vitro* infections. This is another reason for increased reliance on transcriptome and proteome studies, as miRNA signatures, while they can be used as biomarkers of disease status, are perhaps more useful when we can correlate their targets with effects on viral replication. For instance, it is highly probable that these miRNAs may eventually lead to the identification of novel cellular co-factors. Furthermore, the effects of these miRNAs may not be limited to the cell of their origin, as miR-150 has been shown to be secreted in microvesicles, with secretion being upregulated following LPS treatment of human blood cells or of the monocytic cell line THP-1 [124]. MiR-150 secreted from THP-1 cells was uptaken by a recipient endothelial cell line, and expression of the miR-150 target c-Myb was shown to be decreased in response. As Nef appears to dramatically increase microvesicle secretion [125], it is therefore probable that HIV-1 infection may cause changes in expression of secreted miRNAs which may eventually affect cells distributed throughout the periphery.
